# Grain Size-Dependent Thermal Expansion of Nanocrystalline Metals

**DOI:** 10.3390/ma16145032

**Published:** 2023-07-16

**Authors:** Pär A. T. Olsson, Ibrahim Awala, Jacob Holmberg-Kasa, Andreas M. Krause, Mattias Tidefelt, Oscar Vigstrand, Denis Music

**Affiliations:** 1 Materials Science and Applied Mathematics, Malmö University, SE-205 06 Malmö, Sweden; 2 Division of Mechanics, Materials and Components, Lund University, P.O. Box 118, SE-221 00 Lund, Sweden; 3 Division of Solid Mechanics, Lund University, P.O. Box 118, SE-221 00 Lund, Sweden; 4 Biofilms Research Center for Biointerfaces, Malmö University, SE-205 06 Malmö, Sweden

**Keywords:** thermal expansion coefficient, nanocrystalline metals, molecular dynamics modeling, density functional theory modelling

## Abstract

In the present work, we have used classical molecular dynamics and quantum mechanical density functional theory modeling to investigate the grain size-dependent thermal expansion coefficient (CTE) of nanocrystalline Cu. We find that the CTE increases by up to 20% with a gradually decreasing grain size. This behavior emerges as a result of the increased population of occupied anti-bonding states and bond order variation in the grain boundary regions, which contribute to the reduced resistance against thermally-induced bond stretching and dictate the thermal expansion behavior in the small grain size limit. As a part of the present work, we have established a procedure to produce ab initio thermal expansion maps that can be used for the prediction of the grain size-dependent CTE. This can serve as a modeling tool, e.g., to explore the impact of grain boundary impurity segregation on the CTE.

## 1. Introduction

Nanomaterials, usually perceived as artificial or naturally occurring materials with at least one dimension of the order of 1 to 100 nm [1], have made their way into nearly every field of contemporary research. Different types of configurations, such as nanoparticles (3D objects), nanoplates (2D entities, also known as nanosheets or nanoribbons), nanofibers (1D nanotubes, nanorods, or nanowires), and quantum dots (0D configurations) have altered many fundamental physical, chemical, and biological properties of the corresponding parent (bulk) materials [1]. Their unique properties arise when a material’s specific dimension becomes comparable to a characteristic length of some physical property, e.g., the electron mean free path for electrical conductivity [2].

Despite the fact that many fundamental properties of nanomaterials nowadays are well understood after being explored over several decades, there is still an abundance of open questions related to certain properties. On the one hand, nanoscale-induced modifications of several magnetic and mechanical properties have been ratified on the atomic scale. For instance, the magnetic moments of individual atoms drastically increase with decreasing dimensionality, which gives rise to significant magnetoresistance [3] and superparamagnetism [4]. Furthermore, surface-induced variations in the strength and elastic properties of nanosized elements have been widely reported in the literature [5,6,7,8,9,10,11,12,13,14], where the surfaces may promote increased or reduced stiffness depending on the crystallographic orientation. Moreover, polycrystals with nanosized grains preferentially undergo grain sliding rather than dislocation slip to accommodate plastic deformation since it is energetically unfavorable for dislocations to migrate across an increased number of interfaces. This gives rise to an inverse Hall-Petch transition, which has been reported to occur for metals with grain size diameters typically around ∼10–20 nm [15,16,17]. On the other hand, thermal properties are less understood. Such properties include, e.g., thermal conductivity and thermal expansion, which are both known to exhibit grain size-dependent behavior; see, e.g., [18,19,20]. Thermal expansion is an integral part of many intricate thermal features, such as thermal shock, thermal fatigue, and thermal stresses [21,22]. Very often, expansion coefficients are tabulated in textbooks and handbooks without any reference to the factual nature of test materials (single-crystalline or polycrystalline bulk, sintered powder, thin film, or nanomaterials). Hence, different values can often be found for the same material. However, using an inaccurate coefficient of thermal expansion (CTE) for materials in engineering applications can lead to unanticipated high stresses, which can have serious consequences for the integrity of the component in question and ultimately lead to unforseen or premature failure.

Recently, it has become evident that the CTE depends on the grain size (or microstructure), which has a severe effect on nanocrystalline materials with grains of the nanometer size. Owing to the anticipated reduced cohesion of grain boundaries (GBs), most polycrystalline solids are expected to exhibit enhanced thermal expansion as the size of the grains decreases and the GB fraction increases. For instance, the linear CTE of Cr is more than doubled by decreasing the grain size from typical bulk values down to 21 nm [19]. Similar effects have been observed for CrN [19], Cu [20,23,24,25,26], Ni [23,24], Ag [25,27], Ni-P alloys [28], Se [29,30,31], and Pb [30,31]. However, among the published results in the literature, there are also reports of the CTE being independent of the grain size for, e.g., Cr, Pd, Ti, Ni, and Ni-Fe alloys [32,33,34,35] or that it can be either smaller or larger than that of conventional micrometer grain-sized polycrystals [36]. These works highlight the fact that the grain size-dependence of the CTE can be of substantial magnitude and that contradicting trends in the behavior have been reported in the literature.

To explain the unusual behavior of CTE, there are mainly two routes that have been adopted in the literature: (i) formulating a nanocrystalline material model [19] and (ii) employing classical molecular dynamics (CMD) modeling [37,38]. Daniel and coworkers [19] have proposed a nanocrystalline material model for Cr and CrN, in which nanomaterials should be described via atoms in two discrete regions, i.e., grains and GBs. The GBs have been suggested to consist of weakly bonded atoms (crystallite or disordered) compared with those within the grains. The volume fractions of these two components are thus crucial in determining the properties of nanostructured materials. This model is qualitative and appears intuitive, but it has not been quantitively related to direct physical observations. CMD modeling has been carried out for some nanomaterials, including Cu nanowires [37] as well as Cu, Ag, and Ni nanometer-thick thin films [38], but no GBs were included in these particular cases. Hence, there is no attempt to critically appraise these approaches simultaneously and explain the unusual behavior of CTE for polycrystals with nanosized grains using both atomistic and electronic structure perspectives.

In the present work, we investigate the size dependence of the linear CTE of nanocrystalline Cu through a combined CMD and ab initio study. We chose Cu as a benchmark nanoscale system thanks to the existence of newer convincing experimental data in the literature [23,24,25] and well-established, highly accurate CMD interatomic potentials. In this theoretical study, the linear CTE for nanocrystalline Cu with grain sizes ranging from 6 to 25 nm is studied by means of CMD, while density functional theory (DFT) is employed to tackle the borderline cases: bulk and amorphous Cu. Consideration of the latter case is based on the fact that a GB is less ordered and decreasing the grain size would increase the volume of the amorphous fraction, while a single-crystal would represent the other extreme—large-sized polycrystals with negligible impact of GBs. This will be used to describe the CTE of abitrary microstructures based on the model proposed by Daniel and coworkers [19] and to identify the electronic structure origin of the behavior of the linear CTE. Thus, by combining the results from these numerical tools, we provide new insight on the grain size-dependent CTE, which, to the best of the authors’ knowledge, has not been reported previously. Moreover, as part of the paper, we devise a novel procedure to make theoretical predictions of the impact of the grain size on the CTE for a wide range of temperatures. We project that this approach can be applied to systems with impurities segregated at the GBs and therefore can be used as a means to search for suitable alloying elements to optimize the CTE.

The paper is organized as follows: In Section 2, we present the method, in which we detail the adopted CMD and DFT modeling strategies. This is followed by a presentation and discussion of the results in Section 3 and Section 4, respectively. Finally, the key findings are summarized and the conclusions are stated in Section 5.

## 2. Methods

### 2.1. Classical Molecular Dynamics Modelling

For the CMD simulations, we used the open-source LAMMPS software [39,40]. To model the interatomic interaction, we used the embedded atom method (EAM) potential for Cu as parametrized by Mishin et al. [41]. This classical EAM potential is considered to be the state-of-the-art benchmark potential for Cu and exhibits excellent transferability and reproduction of experimental and DFT data. Notably, it has demonstrated itself to be particularly powerful when it comes to predicting accurate phonon data, thermal expansion, and elastic constants for single-crystals [41]. It has also shown good agreement with DFT for GB energies for symmetric tilt 〈110〉 GBs [42]. These features are anticipated to play central roles in predicting the thermal expansion properties of nanocrystalline Cu, which makes this EAM potential highly suitable for the present study.

**Figure 1:**
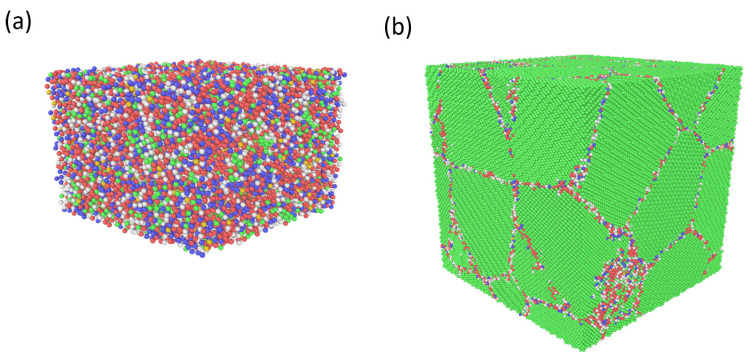
(**a**) An amorphous configuration and (**b**) a nanocrystal comprising 13 grains with the averaged diameter of 12.9 nm. The Cu atoms are colored using the Ackland-Jones characterization approach [49] such that green, red, blue, and grey particles have FCC, HCP, BCC, and unknown coordination, respectively. The images were generated using the OVITO software [50].

Three sets of geometries were generated to investigate the thermal expansion by means of CMD: (i) fully amorphous configurations, (ii) single-crystals and (iii) nanosized polycrystals. To produce fully amorphous structures, we used a multi-step procedure that was initiated by the generation of a randomized structure containing ∼22,000 atoms. It was first heated up to and equilibrated at T=4000 K and zero pressure tensor within the isobaric-isothermal (NPT) ensemble using a Nosé-Hoover thermostat and fully anisotropic barostat [43,44,45,46]. Following this melting procedure, which lasted for 0.75 ns (using a timestep of 0.25 fs), the system was quenched and equilibrated at room temperature such that a non-crystalline structure was obtained; see Figure 1a. The high temperature used in the first step of the process was adopted to create a highly disordered template with particles of high kinetic energy, from which realistic amorphous structures could be obtained during the subsequent cooling process. Periodic atomistic nanocrystals with different grain sizes were generated using the ARL-COMBAT toolkit [47,48], which relies on Voronoi tesselation to produce the polycrystalline geometries. Four different specimens, each containing 13 or 14 grains, with the averaged grain size diameter corresponding to d=6.44,12.9,18.9, and 25.7 nm, respectively, were generated, see Figure 1b.

To compute the thermal expansion of the amorphous configurations and single-crystals, the change in volume was sampled at a variety of temperatures. Owing to the fact that quantum mechanical effects in the low temperature limit, such as zero-point energy, are not captured by CMD modeling, lower temperatures than −100 °C were not considered in any of the classical simulations. Thus, we limited this study to the temperature span ranging from −100 to 525 °C, with an increment of 25 °C, which provided a sufficiently fine resolution of the volume expansion for the present study. The specimens were first equilibrated at the target temperature and zero pressure for 1.0 ns, whereafter the volume was sampled and averaged for an additional duration of 1.0 ns within the NPT ensemble. The generated temperature-dependent volume data was then fitted to a third-order polynomial, from which the linear CTE was computed as

(1) $$\alpha_{L}\left(T\right)=\frac{1}{3}V_{ref}^{-1/3}V{\left(T\right)}^{-2/3}\frac{dV}{dT},$$

where $V_{ref}$ is the reference volume at T=25 °C; see Appendix A.

The same sampling strategy was adopted for the nanocrystalline specimens, with the addition that we also computed and summed up the Voronoi volume associated with each particle in every grain, such that the volume of each grain as well as the total volume could be monitored. The thermally-induced volume expansion was then computed on both an individual grain level and globally by sampling the respective volumes. Owing to the increased computational cost associated with modeling nanocrystals, we limited the investigation to temperatures in the range between −100 to 50 °C with an increment of 25 °C.

### 2.2. Ab Initio Modelling

To estimate the linear CTE of single-crystalline and amorphous Cu, DFT was employed as implemented in the software package OpenMX [51]. A linear combination of localized pseudoatomic orbitals [52] and the generalized-gradient approximation (parametrized by Perdew, Burke, and Ernzerhof (PBE) [53]) were used. The basis function of Cu within a confinement scheme [52,54] was Cu6.0-$s^{2}p^{2}d^{2}$, where 6.0 designates the cut-off radius (in Bohr units) and $s^{2}p^{2}d^{2}$ represents the primitive orbitals (rather than the electronic structure). An energy cut-off of 2040 eV (i.e., 150 Ry) was chosen to attain the total energy precision of ∼10${}^{-5}$ eV at 0 K within a real-space grid of 81×81×81. The amorphous configuration of Cu was obtained by using a liquid-quench algorithm, which is a common procedure in the framework of DFT [55]. A Cu configuration with 108 atoms was melted at 4000 K within 1.0 ps using OpenMX (canonical ensemble, velocity scaling), then quenched and relaxed at 0 K, whereafter it was equilibrated at 300 K for 1.0 ps, before it was finally relaxed again at 0 K. The linear CTE of single-crystalline and amorphous Cu was estimated based on the Debye-Grüneisen model [56].

To gain insight on differences in the bonding nature of crystalline and amorphous Cu, we performed electronic structure calculations to extract the electronic density of states (DOS), for which we used the Vienna ab initio simulation package (VASP) [57,58,59,60]. To elucidate the bonding and anti-bonding state occupancy, the crystal orbital Hamiltonian population (COHP) of atomic pairs within the range of 1.5–3 Å was calculated using the LOBSTER code, a software to analyze chemical bonding based on plane-wave and projector augmented wave (PAW) output [61,62,63,64]. Such analysis is not possible with the basis set used in OpenMX. Moreover, for quantifying differences in the local bonding, we investigate the sum of bond orders associated with each atom by means of the DDEC6 approach as implemented in the CHARGEMOL software [65]. To this end, single point calculations for the crystalline structure were performed on a 25×25×25 *k*-point grid using the tetrahedron method [66], while those for the amorphous structure were adjusted to maintain the same *k*-point density in reciprocal space. The interaction between the valence electrons and the core was described by the PAW method [67,68]. Thus, we used a PAW potential from the VASP library with the electronic valence configuration given by 3$p^{6}$4$s^{2}$3$d^{9}$ and the exchange-correlation functional described within the PBE formalism. A plane-wave energy basis cut-off of 450 eV, along with a convergence criterion for the total energy of 10${}^{-7}$ eV, were used.

## 3. Results

### 3.1. Thermal Expansion of Amorphous and Crystalline Morphologies

**Figure 2:**
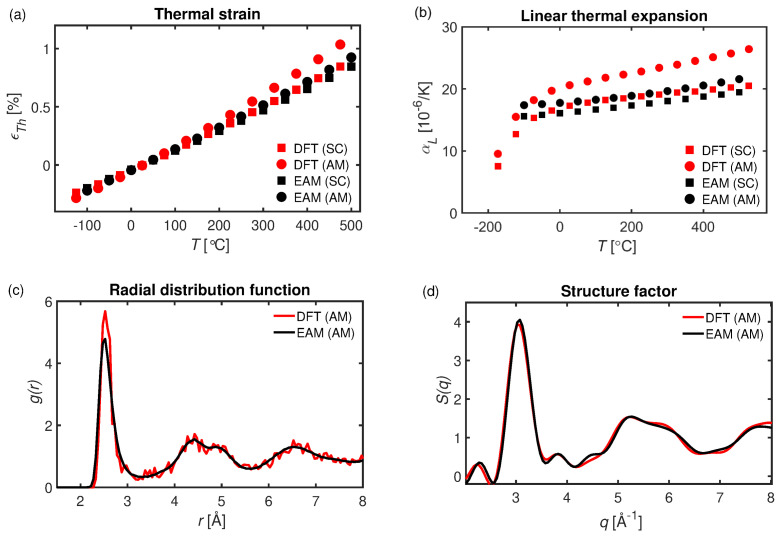
(**a**) Thermally-induced strain for a fully amorphous (AM) configuration and single-crystal (SC) Cu as computed by means of CMD and DFT. Here the reference state corresponds to that at room temperature. (**b**) Temperature dependent CTE for AM and SC. (**c**) Radial distribution function and (**d**) structure factor for AM Cu.

In Figure 2, we have computed the thermally-induced strain, $\epsilon_{Th}$, and the CTE for the amorphous and FCC single-crystalline Cu phases by means of CMD and DFT. For both modeling strategies, it is found that the thermally-induced strain of the amorphous phases exceeds that of the single-crystal FCC phase, but the degree to which they differ varies; see Figure 2a. For the DFT modeling, it is found that $\epsilon_{Th}$ of the amorphous phase is up to 25% higher than the single-crystalline counterpart, while the same variation is only 10% in the classical modeling. This behavior translates to the CTE; see Figure 2b, where it is also observed that the CTE is generally lower for CMD simulations using the EAM potential than for DFT modeling. Specifically, at room temperature, the CTE associated with crystalline Cu is $\alpha_{L}^{SC}=16.22\cdot 10^{-6}$ and $17.30\cdot 10^{-6}$ K${}^{-1}$ for CMD and DFT, respectively. Comparison with experimental data, $∼16.7-17.0\cdot 10^{-6}$ K${}^{-1}$ [69,70], reveals a good agreement with the herein computed data.

The computed CTE for amorphous Cu corresponds to $\alpha_{L}^{AM}=17.86\cdot 10^{-6}$ and $20.60\cdot 10^{-6}$ K${}^{-1}$ for CMD and DFT, respectively, which reveals a difference of approximately 15% for the two approaches. Owing to the fact that the accessible timescale of DFT is significantly less than that of CMD, DFT does not permit elaborate schemes for generating amorphous configurations. Thus, to explore differences in structure, we have computed the radial distribution functions (RDF, see Figure 2c) and structure factor (see Figure 2d) for the amorphous configurations. Although the configuration sizes differ significantly, the particle number densities (80.6 and 81.3 nm${}^{-3}$ for DFT and CMD, respectively) and structure factors are similar. Instead, the most significant difference can be seen in the RDF. It reveals that the average number of neighbors in the first coordination shell is slightly higher for the DFT configuration; see Figure 2c, which could be a contributing factor to the observed discrepancies.

### 3.2. Thermal Expansion of Nanocrystalline Microstructures

**Figure 3:**
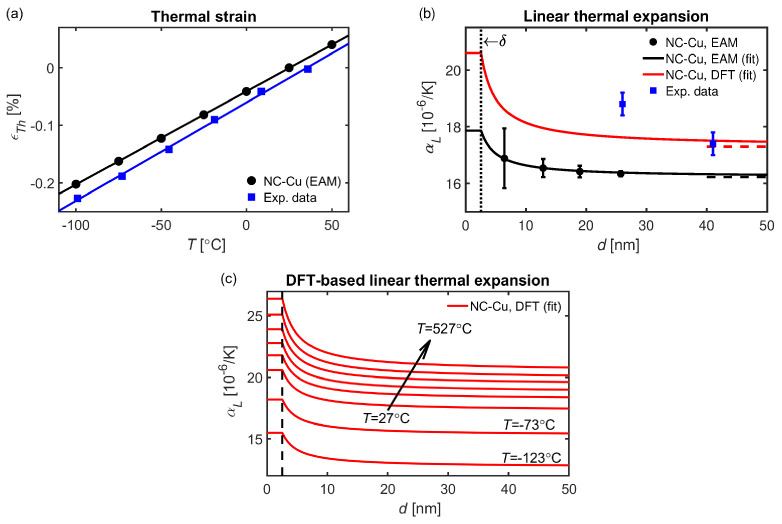
(**a**) Thermally-induced strain for an individual grain as computed by means of CMD for a grain with a d=25.7 nm, along with the experimental data for a microstructure with d=25 nm [23]. (**b**) Thermal expansion at room temperature for nanocrystalline Cu as function of the averaged grain diameter. The vertical error bars correspond to one standard deviation. The vertical dotted line indicates the amorphous/crystalline limit below which the material is considered fully amorphous and the horizontal dashed lines correspond to the linear thermal expansion of single-crystalline Cu as computed by means of CMD and DFT. The experimental data are from [23]. (**c**) CTE map for nanocrystalline Cu as function of the averaged grain diameter for different temperatures, estimated by herein generated DFT data fitted to Equation (2).

The thermally-induced strains for the nanocrystalline microstructures follow an almost linear behavior for the considered temperature range. Such tendencies are in line with the experimental observations in [23], although the slope is steeper than for the CMD results; see Figure 3a. This is indicative of a slight deviation between the computed CTE and experimental data; see Figure 3b, where the former underestimates the room temperature data by 15% for nanocrystalline Cu with d∼25 nm.

The most simplistic approach to estimating the grain-size dependent CTE is to compute the weighted average based on the fraction of grain and interface atoms [19,28]. To achieve such a construction, it is assumed that the grain volume approximately scales as $V_{G}∼d^{3}$. Likewise, it is assumed that the GB volume, $V_{GB}$, is proportional to $\delta \cdot d^{2}$, where δ is related to the GB interface thickness and serves as the lower crystalline length scale limit, below which grains can be considered fully amorphous. Thus, the volume fraction of GB atoms, $f_{GB}=V_{GB}/V_{G}$, is proportional to the ratio δ/d. By weighted consideration of the amorphous and crystalline extremes [19,28], the averaged CTE of nanocrystalline microstructures can be assessed as a function of the average grain diameter

(2) $$\alpha_{L}^{NC}\left(d,T\right)=\frac{\delta }{d}\alpha_{L}^{AM}\left(T\right)+\left(1-\frac{\delta }{d}\right)\alpha_{L}^{SC}\left(T\right),$$

which converges towards the single-crystalline CTE, $\alpha_{L}^{SC}$, in the large grain size limit and the amorphous counterpart, $\alpha_{L}^{AM}$, in the d→δ limit. To approximate δ, we fit Equation (2) to the grain size-dependent CTE at room temperature as computed by means of CMD. From Figure 3b, it is seen that Equation (2) fits the CMD data well, which is an indication that it is a suitable ansatz to describe the grain size-dependent CTE. The optimal fit of the interfacial thickness to the CMD data corresponds to δ≈2.5 nm, which is of the same order as the transmission electron microscopy detection limit for grains embedded in an amorphous matrix [71,72] and the detection limit for metals using conventional X-ray powder diffraction techniques [73]. Compared with experimental data at room temperature [23], the general observation is that the CTE computed by means of CMD is consistently low for the entire considered range of *d*.

By employing the amorphous and crystalline CTE data from the DFT calculations in tandem with the value of δ estimated from CMD simulations, we used Equation (2) to compute an analogous ab initio-based grain-size-dependent CTE curve at room temperature (see Figure 3b). Owing to the higher values of $\alpha_{L}^{SC}$ and $\alpha_{L}^{AM}$ predicted by DFT, the resulting curve is in better agreement with experimental data [23]. For completeness, in Figure 3c, we have further expanded this analysis beyond room temperature by using DFT data for a range of temperatures to provide a theoretical map of the CTE for different temperatures and grain sizes.

### 3.3. Electronic Scale Analysis

**Figure 4:**
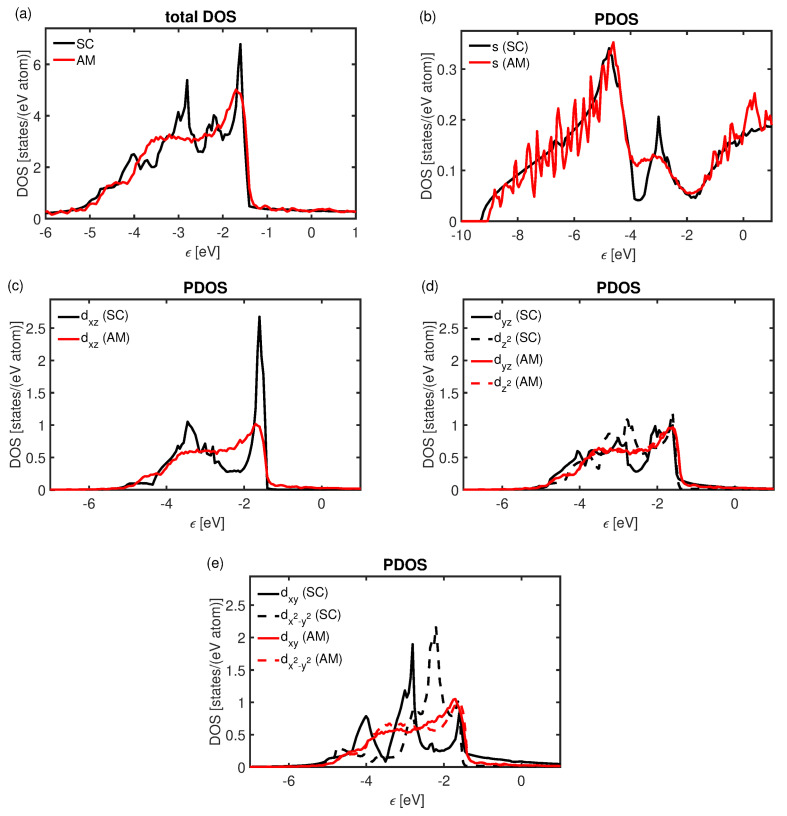
(**a**) Total DOS and (**b**–**e**) projected DOS for single-crystalline and amorphous Cu. The Fermi level is chosen as reference.

In Figure 4, the DOS for crystalline and amorphous Cu can be seen. Most of the states are in the valence band for both configurations, which is in agreement with the earlier studies [74]. As previously reported in studies on other materials [75], the total DOS of both systems is similar but shows some fundamental differences. While the total DOS for crystalline Cu reveals peaks at discrete energy levels; see Figure 4a, that of the amorphous structure is smeared out over an energy range that spans from −5 to −1 eV (relative to the Fermi level). The projected DOS reveals that the *s*-orbitals are only mildly affected by the loss of long-range order in the structure, see Figure 4b. Notably, these states can be found in a broad range between −10 and −4 eV with a smoother distribution in the case of the crystalline structure. The peak of the states at −3 eV is reduced in the amorpous structure. The DOS associated with *d*-orbitals undergo more significant changes. Owing to the random nature of the amorphous phase, the DOS is very similar for all *d*-orbitals, see Figure 4c–e. A broader distribution of states over the energy range between −5 and −1 eV can be observed in the amorphous system, while the crystalline system exhibits localized peaks at discrete energies for all orbitals. The states associated with $d_{xz}$ orbitals are shifted to lower energies in the case of the amorphous structure, while the states of $d_{yz}$ and $d_{z^{2}}$ are more evenly distributed over the energy range. The projected states connected to the $d_{xy}$ and $d_{x^{2}-y^{2}}$ orbitals are shifted towards higher energies. This leaves an overall shift of the states to higher energies for the amorphous configuration. As the *d*-orbitals are intimately connected to the binding of transition metals, such tendencies are indicative of bond strength reduction. Such reductions in bond strength can be derived from the COHP, which implies that more anti-bonding states are populated for the amorphous phase compared with the crystalline structure. Notably, the average integrated COHP yields −0.47 eV for the crystalline configuration, while being −0.23 eV in the amorphous system. Likewise, the bulk modulus is reduced from 140 to 130 GPa upon transition to the amorphous state. These tendencies support the notion that the overall bond strength is reduced in the amorphous counterpart. Owing to the fact that the number of available valence electrons for bond formation per atom is the same for both models, the overall total bond order sum is the same for the crystalline and amorphous configurations, ∼3.09. However, while the standard deviation is zero for the single crystal, it is 0.12 for the amorphous configurations, which suggests that the bonding variation promotes localized regions of reduced bond strength in the disordered region. This is indicative of an overall reduced bond strength, which upon heating would induce locally increased atom separation and thus contribute to a higher CTE for the GB regions of the nanocrystalline microstructures.

## 4. Discussion

In the present work, we have employed CMD modeling with a classical EAM potential to probe the thermal expansion of nanocrystalline Cu. Such modeling can bridge the time and length scales necessary to investigate the relevant properties of microstructures with realistically sized crystallites. Supplemented with ab initio modeling, insight into the underlying electronic scale mechanisms associated with the differences in cohesion of crystallites and GB regions can be gained. These modeling techniques, in tandem with a simple ansatz to describe the separate contributions from the crystalline and amorphous parts to the overall thermal expansion, have enabled us to establish a model that can provide quantitative grain size dependent predictions of the CTE. Other more advanced approaches to achieving such predictive tools exist for nanoscale elements, e.g., by deriving a size-dependent thermodynamic equation of state. Such formulations have been successfully used for, e.g., Ag, Al, Cu, Pb, Ni, Si, Zn, and Se [76,77]. However, they have been applied predominately for nano-objects with well-defined crystallographic free surfaces. Moreover, such equations of state typically require input parameters, such as cluster diameter, number of constituting atoms, and the Anderson-Grüneisen parameter, which limits their applicability for polycrystals with random grain orientations.

The computed $\alpha_{L}^{AM}/\alpha_{L}^{SC}$-ratio, which is a metric for the mismatch of the CTE of crystal and GB, lies in the range 1.10–1.20 at room temperature. Although older measurements for Cu have indicated that it can be as high as 2.5–5.0 [20,26], experimental investigations of other FCC metals have indicated lower ratios (2.1 for Ag [27]) or that no distinction in the CTE of crystal and GB could be observed (Pd [32]). By using the fitted data for δ and the most recent available experimental data in the literature [23,24], we estimate the experimental $\alpha_{L}^{AM}$ at room temperature by fitting to Equation (2). This analysis indicates that $\alpha_{L}^{AM}∼30\cdot 10^{-6}$ K${}^{-1}$, which is of the order of 50% higher than was found by the DFT and CMD modeling. This result would suggest that the experimental $\alpha_{L}^{AM}/\alpha_{L}^{SC}$-ratio is ∼1.8. Thus, more modest thermal expansion mismatches between the interfacial and crystalline regions have also been obtained, which is more in line with our modeling results. These observations indicate that there is significant scatter in the experimental data reported in the literature and that the ability to predict the thermal expansion of nanocrystalline materials hinges on other—possibly overlooked—factors than just the grain size, which can influence the measurement of thermal expansion properties.

As pointed out by Kuru et al. [23], aspects such as the presence of residual stresses that induce severe plasticity and vary with temperature could be a source of error when extracting the thermal expansion data. Moreover, depending on the characterization technique, factors such as porosity, which changes with temperature, can dilute the measured data. The herein simulated nanocrystalline geometries were constructed using Voronoi tesselation with random grain orientations, which produces low-stress and non-textured specimens without any porosities, such that they can be considered idealized microstructures. Furthermore, by adopting an anisotropic barostat during the sampling, the stresses within each grain were maintained low at approximately ±100 MPa, such that no plasticity would emerge. Thus, we anticipate that the herein computed data are unaffected by such factors, which would suggest they represent an idealized situation where high stresses and porosities are absent. Another important aspect is the role of impurity segregation in the GBs. Although typically not considered in the literature, impurities can drastically affect physical properties even at low concentrations. For instance, segregation of oxygen in GBs is expected to impact the local bond strength, which will influence the overall CTE in the small grain size limit. Thus, a lack of impurity control could be a further contributing factor to the discrepancies observed for the herein computed data and experimental observations.

The electronic scale analysis reveals a net shift of occupied *d*-orbitals towards higher energies for the amorphous state, which is accompanied by an increased occupancy of anti-bonding states and scatter in the bond order. Such behavior is indicative of reduced cohesion in the disordered GB regions. These tendencies can be further enhanced by the segregation of weakening species, which would promote increased CTE for the GB regions and ultimately for the overall material. Conversely, GB engineering by alloying bond-strengthening elements could reduce the CTE of GBs to that of the crystallites—or lower—which in principle would render the opposite trend to that observed in Figure 3b,c. Although it lies beyond the scope of the present work, we anticipate that similar ab initio thermal expansion maps as those in Figure 3c could be produced to incorporate the effects of impurity GB segregation merely by considering samples of mixed element types when computing the CTE of the amorphous region. This could be used as an in silico tool to explore suitable alloying elements for optimizing the thermal expansion properties of nanocrystals.

## 5. Summary and Conclusions

In the present work, we have used a combination of CMD and electronic-scale DFT modeling to investigate the thermal expansion of nanocrystalline Cu. The results indicate that the CTE increases with decreasing grain size by up to ∼20% in the small size limit. This behavior is attributed to the reduced cohesion of GBs, which emanates from an increased occupation of anti-bonding states and variation in bond order that weaken the overall bond strength and thereby reduce the resistance to thermally-induced bond stretching. The overall thermal expansion behavior is less pronounced than that observed in experimental studies, where it has been reported that the CTE of GBs can be up to five times higher than the crystalline counterpart, although it is noted that there is significant scatter among the available experimental data. We have discussed possible reasons for the discrepancy between modeling and experimental results.

Finally, by using a straightforward ansatz that relies on a volume-weighted average of the CTEs of the crystalline and amorphous parts, we have established a means to produce ab initio thermal expansion maps that enable the prediction of the grain size-dependent CTE. It is an integral part of many thermal properties, such as thermal shock, which highlights the fact that the results obtained herein are of importance for the thermal management of advanced materials in novel applications.

## Data Availability

The data required to reproduce these findings will be provided by the corresponding author upon reasonable request.

## Appendix A. Linear Thermal Expansion

In the present appendix we derive the used expression for computing the thermal expansion, Equation (1). The linear thermal expansion is defined as

(A1) $$\alpha_{L}=\frac{1}{L_{ref}}\frac{dL}{dT},$$

where *L* is a characteristic length dimension that depends on the temperature and $L_{ref}$ is the reference length. For an isotropic material or one with cubic symmetry, the characteristic length can be chosen as $V^{1/3}$, where *V* is the temperature-dependent supercell volume or the volume of individual grains. Substitution of *L* and $L_{ref}$ with $V^{1/3}$ and $V_{ref}^{1/3}$, respectively, into Equation (A1) yields

(A2) $$\alpha_{L}=\frac{1}{V_{ref}^{1/3}}\frac{d(V^{1/3})}{dT}=\frac{1}{3}V_{ref}^{-1/3}V{\left(T\right)}^{-2/3}\frac{dV}{dT},$$

where $V_{ref}$ is the reference volume at T=25 °C such that the righthand side corresponds to Equation (1). To end up with this result we have used the chain rule to derive the following identity

(A3) $$\frac{dV{\left(T\right)}^{1/3}}{dT}=\frac{dV{\left(T\right)}^{1/3}}{dV}\cdot \frac{dV}{dT}=\frac{1}{3}V^{-2/3}\cdot \frac{dV}{dT}.$$
